# Mad Dog Bites

**Published:** 1874-03

**Authors:** 


### MAD DOG BITES.

Close observation has shown that hydrophobia occurs as often in cold weather as in warm. The bites of other domestic animals, as cats and rats, occasion hydrophobia. Imagination, a highly nervous condition, has been followed by hydrophobic symptoms when no bite of any animal or insect has been remembered; hence, the wisdom in all cases of bites, for the person bitten to do all that is possible to dismiss the subject from the mind; and in case of a child or servant, or other uncultivated person, or any highly nervous person, common sense, philosophy and humanity alike dictate the course to be pursued toward the injured parts. Never show any apprehension, exhibit no misgivings, never allude to the subject in any way ; or, if it is brought up at any time, in mercy to the bitten one turn the conversation in some other direction. The greatest surgeon of the century, having directed his attention to the subject, ascertained that out of twenty persons bitten by dogs apparently mad, only one suffered from hydrophobia.

Mad dogs foam at the mouth and bark severely, because they have been excessively irritated and alarmed. Any dog does the same. True hydrophobic dogs never do foam at the mouth, never do bark ; and yet persons suffering from hydrophobia do imitate the barking of a dog, showing conclusively that they are laboring under a false impression as to facts ; and in such cases the attack may be well considered as much nervous as a fit of hysterics. Such person might be brought to by a warm bath, cold cloths ; or, better still, bags or pads of pounded ice and salt kept applied to the whole head, while the entire body, in a room marking seventy degrees of Fahrenheit, should be immersed in water at ninety-five degrees and kept there until a soothing effect has been obtained, and repeat as often as needed. This is not given as a cure for real or bastard hydrophobia, but as an alleviant of the dreadful sufferings.

After all, what is the sign of a dog being mad, in a hydrophobic sense? Do they run and yelp as if Old Scratch or a tin pan were following close after them? Not a bit of it. They are moody, quiet, restless, uneasy, paw the ear, and will snap at you if you don't let them alone—just as many people snap and snarl when they are not very well, or have the toothache. The very best treatment for a mad dog is precisely that which is the very best for a mad man—to one who has lost his temper and made a fool of himself—let him alone; or, which is more humane as to the dog, get him into a silent retired room, and, once or twice a day, slip in some water and meat. If in two or three days he does not come to know you, by wagging his tail when you call him by name, then it might be as well to destroy him by chloroforming him.

THE SAFEST PLAN.

If a person has been bitten by a dog supposed to be mad—if a physician cannot be had promptly—suck the blood out of the wound vigorously for two or three minutes, if there is no sore or crack about the lips, tongue or mouth; then heat any piece of iron as sharp as the point of a goose quill until it is of a white heat and press it into the wound for half an inch or more, not keeping it in more than five seconds; this is called "cauterizing"—a method which can be made speedily available almost anywhere. If you have not a sharp bit of iron take a live coal of any kind and lay it on the wound, regardless of its greater size, for burning a wider surface will answer the purpose of a deeper one; let the live coal remain on ten seconds. The treatment, if promptly done, is believed by eminent men to be a certain and perfect preventive in all cases.

